# Combination of sST2/LVMI Ratio and Modified MELD Scores Predicts Mortality in End-Stage Heart Failure

**DOI:** 10.3390/ijms26010171

**Published:** 2024-12-28

**Authors:** Wioletta Szczurek-Wasilewicz, Michał Jurkiewicz, Michał Skrzypek, Ewa Romuk, Jacek Jóźwiak, Mariusz Gąsior, Bożena Szyguła-Jurkiewicz

**Affiliations:** 1 2nd Department of Cardiology and Angiology, Silesian Center for Heart Diseases, 41-800 Zabrze, Poland; 2 Department of Pharmacology, Faculty of Medicine, University of Opole, 45-040 Opole, Poland; 3 Student’s Scientific Society, 3rd Department of Cardiology, Faculty of Medical Sciences in Zabrze, Medical University of Silesia, 40-007 Katowice, Poland; 4 Department of Biostatistics, Faculty of Public Health in Bytom, Medical University of Silesia, 40-007 Katowice, Poland; 5 Department of Biochemistry, Faculty of Medical Sciences in Zabrze, Medical University of Silesia, 40-007 Katowice, Poland; 6 Department of Family Medicine and Public Health, Faculty of Medicine, University of Opole, 45-040 Opole, Poland; 7 3rd Department of Cardiology, Faculty of Medical Sciences in Zabrze, Medical University of Silesia, 40-007 Katowice, Poland

**Keywords:** heart failure, markers, risk stratification

## Abstract

Biomarkers are critical for heart failure (HF) management by facilitating risk stratification, therapeutic decision-making, and monitoring treatment response. This prospective, single-center study aimed to assess predictors of death during one-year follow-up in patients with end-stage HF, with particular emphasis on the soluble suppression of tumorigenicity 2/left ventricular mass index (sST2/LVMI) ratio, modified Model for End-stage Liver Disease (modMELD), and Model for End-stage Liver Disease excluding INR (MELD-XI). This study comprised 429 consecutive patients with end-stage HF hospitalized between 2018 and 2023. The median age was 56.0 (50.0–60.0) years; and 89.2% were male. During the follow-up, 134 (31.2%) patients died. The area under the receiver operating characteristics (ROC) curves showed good predictive powers of sST2/LVMI-MELDXI (AUC: 0.90 [CI: 0.87–0.93]; specificity 85% and sensitivity 80%) and sST2/LVMI-modMELD (AUC: 0.92 [95% CI: 0.90–0.95]; specificity 92%, sensitivity 81%) for assessment of one-year mortality. In conclusion: the sST2/LVMI-modMELD and sST2/LVMI-MELD-XI ratios are independently related to one-year mortality in the analyzed group of patients. The prognostic power of these new models is significantly better than their individual components. This single-center study comprised a relatively small group of patients, so the prognostic value of these new models cannot be generalized to the entire HF population. Considering the limitations of this analysis, further randomized trials with a large cohort are necessary to confirm the utility of the new prognostic models in HF patients.

## 1. Introduction

Despite advances in pharmacological and interventional treatment, heart failure (HF) poses a serious clinical, social, and economic problem due to its high morbidity and mortality. An important element of HF management is appropriate risk stratification. Prognostic scales have been developed to predict outcomes in patients with HF, but these scales require complex formulas for risk assessment or include additional variables that are not commonly used. In addition, some prognostic models in HF such as Heart Failure Survival Score (HFSS) or Seattle Heart Failure Model (SHFM) were derived and validated in the 1990s and early 2000s. Therefore, it is necessary to create new prognostic models which will facilitate accurate assessment of prognosis and selection of patients requiring advanced interventional and surgical treatment methods [1,2,3,4]. Over the years, there has been a growing interest in searching for new risk stratification models in HF based on readily available simple biomarkers related to the pathophysiology of HF [1]. Among these biomarkers, soluble suppression of tumorigenicity 2/left ventricular mass index (sST2/LVMI) and models for end-stage liver diseases deserve attention [5,6]. The sST2/LVMI ratio reflects two important pathways of HF [6]. sST2 plays an important role in the fibrotic response to inflammation [5]. In turn, LVMI is associated with the prefibrotic inflammatory phase of HF [6]. Other important indicators associated with HF are the models for end-stage liver disease (MELD), which are calculated based on bilirubin and creatinine concentrations in the case of the MELD excluding INR (MELD-XI) scale, and albumin concentration in the case of the modified MELD (modMELD) scale [4]. Those scales reflect cardiorenal and cardiohepatic interactions in HF and higher MELD-XI and modMELD scores are indicators of HF progression and worse outcomes [4,7,8].

This study aimed to evaluate the predictive power of combined sST2/LVMI-modMELD and sST2/LVMI-MELD-XI scores in the patients with end-stage HF during a one-year follow-up. We also searched for other predictors of outcomes in the analyzed cohort.

## 2. Results

The analyzed population included 429 HF patients (New York Heart Association (NYHA) functional classes III or IV, INTERMACS 4–6 profiles). The characteristics of the included patients are shown in Table 1. A total of 134 (32.1%) patients died over the study period.

Table 2 presents the results of the univariable and multivariable analysis. In the first model, sST2/LVMI-MELD-XI, fibrinogen, uric acid, sodium and N-terminal pro-B-type natriuretic peptide (NT-proBNP) were independent risk factors of one-year mortality. The Harrell concordance index for the first final Cox regression model was 0.854. In the second model, sST2/LVMI-modMELD, fibrinogen, uric acid and NT-proBNP were strongly related to one-year mortality. The Harrell concordance index for the second final Cox regression model was 0.861.

In the ROC curves analyses, sST2/LVMI-MELD-XI had a comparable area under curve to that of sST2/LVMI-modMELD (0.900 vs. 0.924, respectively). The Hosmer–Lemeshow test showed appropriate calibration of sST2/LVMI-MELD_XI (*p* = 0.8620) and sST2/LVMI-modMELD (0.6327). The differences between the AUC for the combined scores and the AUC for their components were significant (*p* < 0.05).

**Figure 1:**
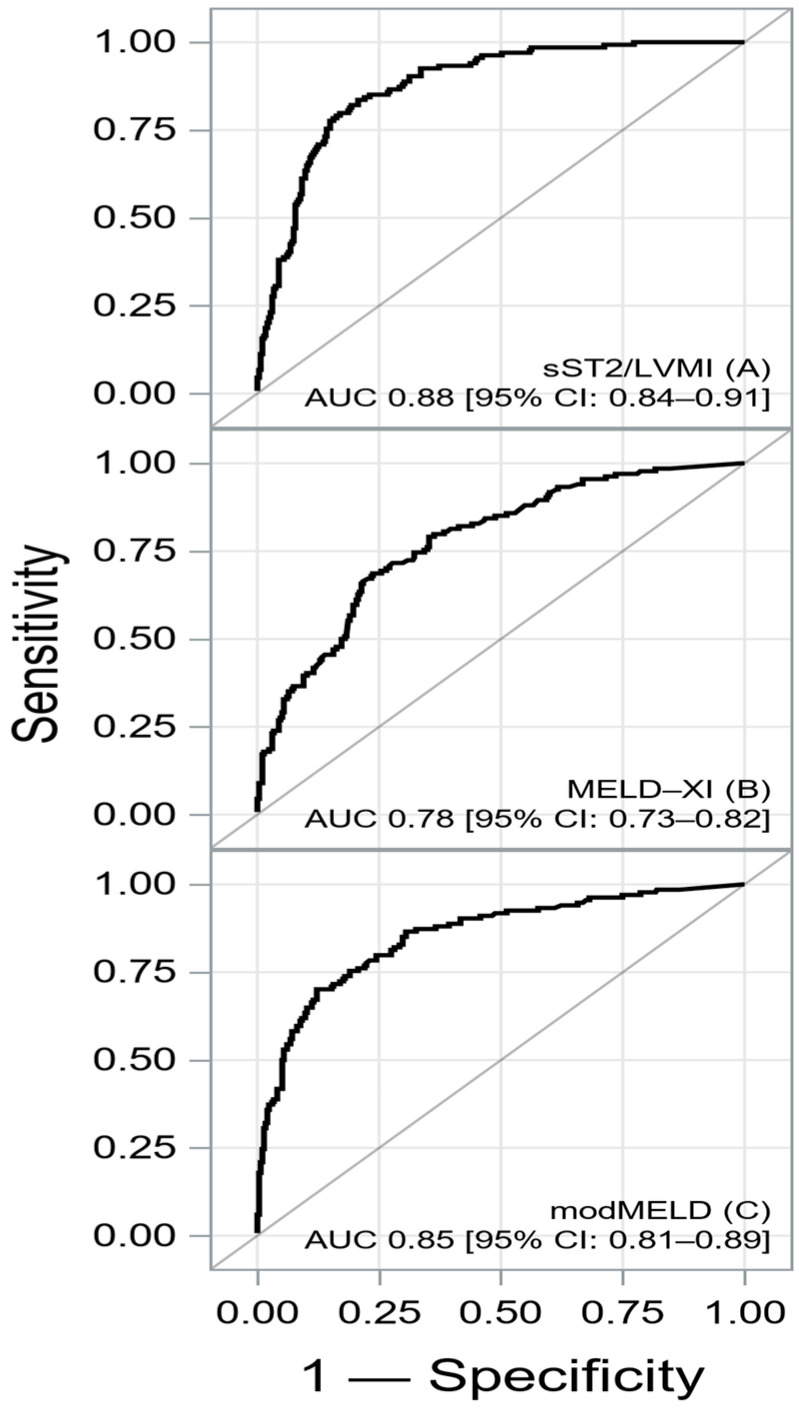
The ROC curves for sST2/LVMI (**A**), MELD-XI (**B**) and modMELD (**C**). Abbreviations: see Table 1.

Receiver operator characteristic (ROC) curves for sST2/LVMI, and MELD scores are presented in Figure 1. 

**Figure 2:**
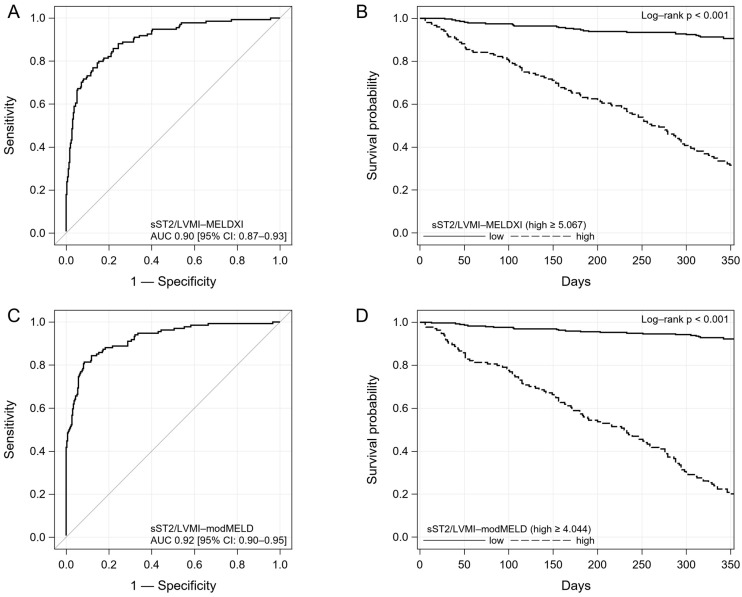
The ROC and Kaplan–Meier curves for sST2/LVMI-MELD-XI (**A**,**B**) and sST2/LVMI-modMELD (**C**,**D**) A summary of the ROC analysis is shown in Table 3.

The ROC curves and Kaplan–Meier survival curves dichotomized according to the cut-off of new models are shown in Figure 2.

**Table 3:** A summary of the ROC curve analysis for biomarkers.

	AUC [±95 CI]	Cut-Off	Sensitivity [±95 CI]	Specificity [±95 CI]	Accuracy
sST2/LVMI	0.88 [0.84–0.91]	≥0.306	0.84 [0.76–0.89]	0.79 [0.74–0.84]	0.81 [0.77–0.84]
MELD-XI	0.78 [0.73–0.83]	≥13.96	0.69 [0.60–0.76]	0.76 [0.71–0.81]	0.74 [0.69–0.78]
ModMELD	0.85 [0.81–0.89]	≥12.55	0.70 [0.62–0.78]	0.88 [0.84–0.91]	0.82 [0.78–0.86]
sST2/LVMI-MELDXI	0.90 [0.87–0.93]	≥5.07	0.80 [0.72–0.86]	0.85 [0.80–0.89]	0.83 [0.79–0.87]
sST2/LVMI-modMELD	0.92 [0.89–0.95]	≥4.04	0.81 [0.74–0.88]	0.92 [0.88–0.94]	0.88 [0.85–0.91]

Abbreviations: see Table 1, AUC—area under the curve, CI, confidence interval.

A comparison of the areas under the ROC curves for the new scores and their components are demonstrated in Table 4.

## 3. Discussion

Our study is the first one to demonstrate that new prognostic scales—sST2/LVMI-modMELD and sST2/LVMI- MELD-XI—are significantly associated with outcomes in analyzed cohort. Both scores have good prognostic powers, sensitivities and specificities, allowing for appropriate risk stratification in the analyzed group of patients. The prognostic power of new models is significantly better than their individual components.

The sST2/LVMI ratio allows for the assessment of two major pathways of HF pathophysiology—inflammation and fibrosis [6]. sST2 is a decoy receptor that inhibits the beneficial cardioprotective effects of Interleukin-33, thus contributing to cardiac hypertrophy, myocardial fibrosis, and in the final phase to HF. sST2 increases the production of inflammatory and pro-oxidative markers in the heart, and measurement of sST2 is useful for assessing the severity of HF and outcomes [6,9,10]. In turn, LVMI allows for the assessment of the prefibrotic inflammatory phase of HF and is a well-established parameter that can independently predict adverse cardiovascular events and premature death [11]. Thus, the combined evaluation of sST2 and LVMI indices allows for an accurate assessment of fibrotic and inflammatory abnormalites in the heart. Our study showed that the sST2/LVMI ratio has good prognostic power, specificity and sensitivity for assessment of outcomes in the analyzed cohort. Only one study by Li also showed the predictive utility of this model in patients with end-stage HF. In this research, an elevated baseline sST2/LVMI ratio was related to an increased risk of short-term outcomes and rehospitalization in HF patients [6].

Similarly to previous analyses [8,12,13], MELD XI and modMELD scores also provided important prognostic information and allowed for accurate assessment of one-year outcomes in the patients with end-stage HF. MELD and its modifications reflect multiorgan dysfunction associated with congestion in the course of HF [14]. The traditional MELD score is calculated based on bilirubin, creatinine and international normalized ratio (INR) levels. However, anticoagulants treatment affects the INR; therefore, modified versions of MELD scales were used—modMELD, in which the INR was replaced by the albumin level and MELD-XI, calculated solely on the basis of bilirubin and creatinine levels [4]. Bilirubin is used to assess the metabolic function of the liver, while albumin allows for the assessment of the synthesizing function of the liver. In turn, the creatinine concentration reflects the severity of kidney failure [12,14,15,16]. Previous studies have shown that liver and kidney dysfunction are important independent risk factors of worse outcomes in end-stage HF [15,16]. However, MELD score and its modifications are better markers of liver and kidney dysfunction than individual laboratory assays, because they allow for a holistic assessment of peripheral organ dysfunction secondary to HF [8,12,13].

Considering the importance of inflammation and fibrosis in HF assessed by LVMI/sST2 and cardio-hepatic and cardio-renal interactions using MELD-XI and modMELD scores, we decided to evaluate the predictive value of the combined model. It seems that the new prognostic models—sST2/LVMI-MELD-XI and sST2/LVMI-modMELD—may better stratify the risk of death by considering several pathological mechanisms of HF and important risk factors, and thus facilitate appropriate decisions regarding HF therapy.

Another finding of our analysis was the association between higher fibrinogen concentrations and lower survival in the analyzed group of patients. Fibrinogen is a glycoprotein complex produced by the liver which participates in processes of coagulation and inflammation [17,18]. Fibrinogen is converted into fibrin monomer under the influence of thrombin, then fibrin cross-links with platelets, increases blood viscosity and contributes to the formation of a clot [19]. Fibrinogen production gradually increases in response to chronic, low-grade inflammatory processes [20]. It is also directly involved in the processes closely related to HF pathogenesis by inducing endothelial dysfunction, stimulating smooth muscle cells proliferation and migration and increasing chemotaxis of leukocytes, monocytes and fibroblasts [20,21,22,23,24]. Thus, fibrinogen as a marker of low-grade inflammation and thrombosis may be linked to the development and progression of HF [18,20]. However, studies on the predictive value of fibrinogen level in HF patients are few [21,22,23,25]. Cugno showed that HF leads to hypercoagulability and increased fibrinogen concentration, which may play a key role in thromboembolic complications of HF [21]. Another study also showed that higher fibrinogen concentrations (≥284 mg/dL) are strongly related to worse outcomes in the patients with acute exacerbation of chronic HF [22]. The analysis by Yang also demonstrated that a higher fibrinogen to bilirubin ratio is independently associated with mortality in HF patients [23]. In turn, the analysis by Xu showed a nonlinear relationship between baseline fibrinogen level and the rehospitalization risk in HF patients within 6 months [25]. However, in this study, the authors did not find the relationship between fibrinogen concentration and the risk for death during the follow-up [25].

Our analysis also demonstrated the utility of commonly known predictors of death. Lower sodium concentration as well as an elevated NT-proBNP and uric acid levels predicted higher risk of one-year mortality in the analyzed population. Hyponatremia is a common abnormality in HF patients with a prevalence ranging from 11 to 27%. According to previous analyses, persistent hyponatremia is a strong predictor of worse prognosis in the patients with HF [26,27]. From the pathological point of view, in the early stages of HF, the expansion of extracellular fluid volume is caused by sodium and water retention by the kidneys, which contributes to the appearance of peripheral edema with the progression of HF. As HF progresses, there is a gradual impairment of water excretion by the kidneys, and an increase in antidiuretic hormone causes an aquaristic defect, which, in combination with diuretics treatment, leads to hyponatremia [26]. In addition, low cardiac output in end-stage HF contributes to activation of the renin–angiotensin–aldosterone system (RAAS) and increased angiotensin II concentration, which is a strong thirst stimulant, resulting in increased water intake by the patient [26].

Similarly to low sodium concentration, elevated NT-proBNP concentration is a commonly known prognostic factor in patients with advanced HF [28,29,30,31]. Natriuretic peptides are the most widely studied and used in clinical practice to aid the diagnosis of HF, and assess the effect of therapy and prognosis in patients with HF [30,31]. They play an important regulatory role in responding to myocardial stretch and cause a decrease in sympathetic nervous system expression, increase diuresis, reduce peripheral resistance, and increase smooth muscle relaxation [29,30,31]. Despite the importance of NT-proBNP in assessing the prognosis in HF, it should be emphasized that many factors affect the concentration of natriuretic peptides, including age, anemia, renal failure, atrial fibrillation, hyperthyroidism or obesity [32]. Therefore, the NT-proBNP values should be interpreted taking into account comorbidities and clinical conditions affecting them.

The last independent factor related to one-year mortality in the analyzed group of patients was higher uric acid concentration. Many previous studies showed that hyperuricemia is a common abnormality in HF and is an important factor of worse outcomes [33,34]. Furthermore, higher uric acid concentration is related to increased natriuretic peptides concentration, increased ventricular filling pressure, worse peak oxygen uptake and lower cardiac output; thus, it may reflect the severity of HF [35,36]. The importance of uric acid in HF is related to the induction of inflammation in endothelial and smooth muscle cells, as well as increased production of free oxygen radicals (ROS) [37,38]. Uric acid is the end-product of purine metabolism produced by xanthine oxidase, which converts hypoxanthine into xanthine and xanthine into uric acid [39]. In hypoxic, catabolic or inflammatory conditions which are presented in HF, xanthine oxidase is activated, which contributes to increased production of ROS [40]. In turn, disturbances of the oxidative–antioxidative balance towards pro-oxidation leads to endothelial dysfunction and activation of inflammation, which are closely related to the HF pathophysiology [40]. Hyperuricemia also inhibits myocardial cells activity by activating the extracellular signal-regulated kinase pathway and induces cardiomyocyte apoptosis by activating calpain-1, thus leading to damage to the myocardium [1,41]. Increased uric acid concentration further activates the renin–angiotensin–aldosterone system, which is one of the most important pathways in the pathophysiology of HF [42]. Furthermore, other comorbidities such as renal failure and treatment with loop diuretics and potassium-sparing diuretics constitute an additional source of elevated serum uric acid in HF patients [35].

Over the years, many biomarkers have been identified that reflect different pathways of HF, each with usefulness and a set of limitations. Due to the complex pathophysiology of HF, it seems that a multimarker approach better reflects the nature of HF and allows for more accurate risk stratification [1,2,3,5]. However, it should be emphasized that new biomarkers such as ST2, IL-33, adrenomedullin, copeptin, GDF-15 and galectin-3, despite promising results, have not been included in the European Society of Cardiology guidelines as an element of HF management [1,5]. Further prospective multicenter studies are necessary to verify the utility and clinical relevance of currently studied biomarkers in HF.

### Limitations of the Study

This study included a relatively small population and data were collected from a single center. Therefore, the results obtained could not be addressed to the whole population of patients with HF. Furthermore, our study did not have a validation group. Our analysis required many exclusion criteria, which is also a limitation of this study. Further studies are needed to evaluate the usefulness of new models in the population of HF patients undergoing interventional and surgical treatment. We only collected the data of the patients at the time of inclusion to this study. Furthermore, this study was conducted at the time when guidelines for HF treatment were changing. Some participants were included in this study before the era of SGLT2 inhibitors treatment for HF. It is not known whether there were differences in SGLT2 therapy in survival and nonsurvival groups during the follow-up period, which could potentially affect the presented results. However, it should be emphasized that there were initially no differences between the analyzed groups in terms of pharmacological treatment, and more than half of the patients were initially taking SGLT2 inhibitors, despite the fact that this study was conducted in 2018–2023, and SGLT2 in HF therapy was recommended in the 2021 guidelines for HF. Further studies should be conducted in a large population of the patients treated with SGLT2 inhibitors. In addition, pharmacological therapy was modified depending on the patient’s clinical condition. Thus, the treatment effect may potentially influence the study end-point. To confirm the utility of the new models in HF patients, further multicenter analyses with a large population of patients and external validation are necessary.

## 4. Materials and Methods

### 4.1. Study Population

**Figure 3:**
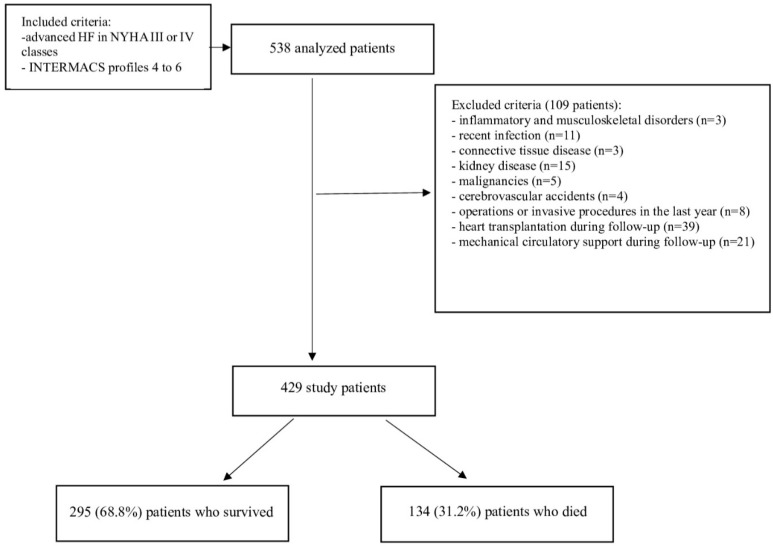
The scheme of this study. Abbreviations: HF, heart failure, INTERMACS, Interagency Registry for Mechanically Assisted Circulatory Support, NYHA, New York Heart Association.

This was a single-center prospective study of the patients with end-stage HF hospitalized in our department between 2018 and 2023. The study design is shown in Figure 3. The end-point of the study was one-year mortality. During hospitalization, each patient underwent a medical history review, a physical examination, baseline laboratory tests, echocardiography, ergospirometric test, and right heart catheterization on admission. All included patients received recommended treatment for HF according to the European Society of Cardiology guidelines at least 3 months before inclusion to the study [43]. The Medical University of Silesia ethics committee approved the study protocol (specific ethics codes: KNW/0022/KB1/53/18, data of approval: 19 June 2018 and PCN/0022/KB1/20/I/21, data of approval: 4 January 2021). All patients signed written informed consent.

### 4.2. Echocardiography

Transthoracic echocardiography examinations were performed by experienced echocardiographers using a Philips Sonos 7500 (Philips Medical Systems, Eindhoven, The Netherlands). Conventional imaging techniques using typical projections, pulsatile and continuous wave Doppler, tissue Doppler, and 3D images were used.

To calculate LV mass (LVM), the following parameters were measured: interventricular septal thickness at end-diastole (IVSd), posterior wall thickness at end-diastole (PWTd), LV internal dimensions at end-diastole (LVIDd), and LV internal dimensions at end-systole (LVIDs). All dimensions were measured three times and the results were averaged to reduce bias.

The following formulas were used:

LVM (g) = 0.8 × 1.04 × [(LVIDd + IVSTd + LVPWTd)3 − (LVIDd)3] + 0.6).

LVMI = LVM/BSA [mL/m^2^]

### 4.3. Analyzed Biomarkers and Scores

Blood samples from peripheral venous blood were drawn after 12 h of fasting at hospital admission. The hematological and biochemical measures, as well as human sST2 concentrations, were determined as we described earlier [8]. sST2/LVMI was calculated as the ratio of sST2 to LVMI.

We used the modified MELD score to omit the influence of vitamin K antagonists on the INR level.

The MELD XI: (5.11 × ln bilirubin, in mg/dL) + (11.76 × ln creatinine, in mg/dL) + 9.44 [44].

The modMELD score [12]: 1.12 × (ln 1) + 0.378 × (ln total bilirubin, in mg/dL) + 0.957 × (ln creatinine, in mg/dL) + 0.643, if the plasma albumin level was higher than 4.1 g/dL.

The modMELD score: 1.12 × (ln [1 + 4.1 − albumin)]) + 0.378 × (ln total bilirubin, in mg/dL) + 0.957 × (ln creatinine, in mg/dL) + 0.643, if the plasma albumin level was less than 4.1 g/dL.

The raw score for modMELD was multiplied by 10.

A lower limit was 1.0 for all variables and the upper limit for creatinine was capped at 4.0 mg/dL. There was no upper limit for bilirubin and albumin levels.

New scores were created to assess the prognostic utility of combined sST2/LVMI-MELD-XI and sST2/LVMI-modMELD. Both variables (sST2/LVMI and MELD-XI and sST2/LVMI and modMELD separately) were included into the Cox regression model as continuous variables. Both variables were multiplied by the associated β coefficient, and risk scores were calculated as follows:

sST2/LVMI-MELD-XI = 0.241 × MELD-XI + 5.058 × sST2/LVMI

sST2/LVMI-modMELD = 0.206 × modMELD + 4.380 × sST2/LVMI

### 4.4. Statistical Analysis

SAS software, version 9.4 (SAS Institute Inc., Cary, NC, USA) was used to perform the statistical analysis. Continuous variables were expressed as median (upper and lower quartiles) and comparisons between groups were made using the Mann–Whitney U test. Categorical variables were presented as percentages and compared using the chi-square test. The areas under the receiver operating characteristic (ROC) were created to examine the utility of new models. The Youden criterion was used to determine the optimal cut-off value for new scores. Diagnostic utility of each analyzed parameter was evaluated using area under curve, sensitivity, specificity, and accuracy. The Hosmer–Lemeshow goodnes of fit test was calculated for all parameters. To compare the survival rate in patients dichotomized according to the cut-off values of the ROC curves for the new models, Kaplan–Meier curves with the log-rank test were performed. The univariable Cox proportional analysis was used to identify potential indicators of higher risk of death for inclusion in the multivariable analysis. Correlations between factors were assessed by the Spearman rank correlation coefficient. Tolerance and variance inflation factors were used to assess multicollinearity. Because sST2/LVMI-MELD-XI and sST2/LVMI-modMELD were highly correlated (r = 0.94), we created two models including significant clinical and statistical parameters and sST2/LVMI-MELD-XI or sST2/LVMI-modMELD. The results were presented as hazard ratios (HRs) with a corresponding 95% confidence interval (95% CI). A *p* value < 0.05 was recognized as statistically significant.

## 5. Conclusions

This study evaluated noninvasive and simple indicators related to worse outcomes in end-stage HF patients. sST2/LVMI, MELD-XI and modMELD have good prognostic powers and specificities, as well as acceptable sensitivities for prediction of outcomes. New prognostic scales—the sST2/LVMI-modMELD and sST2/LVMI-MELD-XI, which are a combination of the MELD scales and sST2/LVMI—are significantly related to worse outcomes in the analyzed cohort. Both new models have good predictive powers, sensitivities and specificities, allowing for an effective separation of survivors from nonsurvivors during a one-year follow-up. The prognostic power of new models is significantly better than their individual components. Other independent risk factors of worse prognosis were higher levels of fibrinogen, NT-proBNP and uric acid, and lower sodium levels. Considering the above limitations of our study, further prospective analyses with a larger cohort and validation sample are necessary to verify the clinical utility of the new prognostic models in HF. The present study can have clinical significance as it provides relatively simple risk stratification models that take into account commonly measured biochemical and echocardiographic indicators in HF patients. These models may potentially facilitate estimation of worse outcomes and selection of those patients at highest risk who require interventional and surgical methods of the treatment.

## Figures and Tables

**Table 1:** Clinical characteristics of the analyzed population.

	Overal *n* = 429	Survival *n* = 295	Nonsurvival *n* = 134	*p*
Age, years	56. 0 (50.0–60.0)	56 (50.0–60.0)	55 (50.0–60.0)	0.6835
Male, *n* (%)	376 (87.6)	263 (89.2)	113 (84.3)	0.1593
Ischemic etiology of HF (%) HF, *n* (%)	280 (65.3)	191 (64.7)	89 (66.4)	0.736
BMI, kg/m^2^	25.9 (23.1–29.4)	26.1 (23.0–29.7)	25.6 (23.2–28.2)	0.1094
Hypertension, *n* (%)	208 (48.5)	146 (49.5)	62 (46.3)	0.5359
AF, *n* (%)	223 (52)	155 (52.5)	68 (50.7)	0.73
Type 2 diabetes, *n* (%)	234 (54.5)	152 (51.5)	82 (61.2)	0.0620
WBC, ×10^9^/L	7.3 (6.1–8.7)	7.3 (6.0–8.6)	7.4 (6.2–8.7)	0.4293
Hemoglobin, mmol/L	8.8 (8.2–9.6)	8.8 (8.2–9.6)	8.8 (8.1–9.7)	0.902
Uric acid, µmol/L	441.0 (364.0–533.0)	419.0 (343.0–498.0)	502.0 (430.0–599.0)	<0.0001 *
Urea, µmol/L	8.1 (6.0–12.2)	7.7 (5.9–11.7)	9.10 (6.3–12.8)	0.0485 *
Creatinine, umol/L	109.0 (95.0–127.0)	103.0 (90.0–119.0)	126.0 (108.0–138.0)	<0.0001 *
Sodium, mmol/L	139.0 (136.0–140.0)	139.0 (138.0–141.0)	137.0 (135.0–139.0)	<0.0001 *
Total bilirubin, µmol/L	18,7 (12.6–24.4)	16.9 (11.7–22.8)	22.9 (15.9–32.3)	<0.0001 *
Albumin, g/L	42.0 (37.0–45.0)	43.0 (41.0–46.0)	37.0 (35.0–41.0)	<0.0001 *
ALP, U/L	79.0 (61.0–102.0)	77.0 (57.0–101.0)	83.5 (65.0–109.0)	0.0447 *
GGTP, U/L	73.0 (35.0–124.0)	62.0 (33.0–112.0)	91.0 (48.0–144.0)	0.0001 *
Fibrinogen, mg/dL	380.0 (308.0–459.00)	369.0 (292.0–441.0)	412.5 (341.0–537.0)	<0.0001 *
hs-CRP, mg/L	4.2 (1.8–7.0)	3.0 (1.5–5.6)	6.0 (3.8–8.5)	<0.0001 *
NT-proBNP, pg/mL	4095.0 (1955.0–7080.0)	3564.0 (1761.0–6682.0)	5526.0 (2517.0–7645.0)	0.0006 *
sST2, ng/mL	43.2 (33.9–77.9)	35.6 (29.8–45.4)	89.3 (70.2–101.3)	<0.0001 *
VO_2_max, mL/kg/min	11.1 (10.2–11.9)	11.0 (10.1–11.8)	11.2 (10.3–12.0)	0.0618
CI, L/min/m^2^	1.9 (1.7–2.0)	1.9 (1.7–1.9)	1.9 (1.7–2.1)	0.7124
LVEDd, mm	74.0 (69.0–79.0)	73.0 (68.0–78.0)	75.5 (70.0–82.0)	0.0014 *
IVSd, mm	10.0 (9.00–11.0)	10.0 (9.0–11.0)	10.0 (9.0–11.0)	0.2246
PWTd, mm	10.0 (9.0–11.0)	10.0 (9.0–11.0)	10.0 (9.0–11.0)	0.2405
LVEF, %	18.0 (15.0–20.0)	18.0 (15.0–21.0)	18.0 (15.0–20.0)	0.1898
LVMI, g/m^2^	176.7 (151.3–205.9)	174.0 (149.9–199.2)	189.5 (160.5–226.5)	0.0002 *
Therapy on admission, *n* (%)				
ACEI/ARB, *n* (%)	398 (92.8)	272 (92.2)	126 (94)	0.4983
B-blockers, *n* (%)	400 (93.2)	273 (92.5)	127 (94.8)	0.3931
Loop diuretics per os, *n* (%)	429 (100)	295 (100.0)	134 (100.0)	1.00
MRA, *n* (%)	406 (94.6)	282 (95.6)	124 (92.5)	0.1928
Empagliflozin/dapagliflozin, *n* (%)	223 (52)	155 (52.5)	68 (50.7)	0.73
ICD/CRT-D, *n* (%)	429 (100)	295 (100.0)	134 (100.0)	1.00
Statins, *n* (%)	307 (71.6)	214 (72.5)	93 (69.4)	0.5041
VKA/NOAC, *n* (%)	223 (52)	155 (52.5)	68 (50.7)	0.73
Other parameters				
ModMELD	10.2 (8.0–13.2)	9.0 (7.5–11.1)	14.2 (11.7–16.9)	<0.0001 *
MELD-XI	12. 8 (10.9–15.1)	12.0 (10.5–13.9)	14.8 (13.3–17.1)	<0.0001 *
sST2/LVMI	0.26 (0.19–0.40)	0.22 (0.16–0.29)	0.45 (0.34–0.58)	<0.0001 *
**sST2/LVMI-MELD-XI**	**4.5 (3.8–5.6)**	**4.1 (3.6–4.7)**	**5.9 (5.3–6.8)**	<0.0001 *
**sST2/LVMI-modMELD**	**3.4 (2.7–4.5)**	**2.9 (2.4–3.5)**	**4.9 (4.2–5.7)**	<0.0001 *

The data are presented as medians (25th–75th percentiles) or numbers (percentages) of patients. * *p* < 0.05 (statistically significant). Abbreviations: ACEI, angiotensin-converting enzyme inhibitor; AF, atrial fibrillation; ALP, alkaline phosphatase; ARB, angiotensin receptor blocker; BMI, body mass index; CI, cardiac index; CRT-D, cardiac resynchronization therapy-defibrillator; GGTP, gamma-glutamyl transpeptidase; HF, heart failure; hs-CRP, high-sensitivity C-reactive protein; ICD, implantable cardioverter-defibrillator; IVSd, septal thickness at end-diastole; LA, left atrium; LVEDd, left ventricular end-diastolic dimension; LVEF-left ventricular ejection fraction; LVMI, left ventricular mass index; MELD-XI, Model for End-stage Liver Disease excluding INR; modMELD, modified Model for End-stage Liver Disease; MRA, mineralocorticoid receptor antagonist; NOAC, Non-vitamin K antagonist oral anticoagulant; NT-proBNP, N-terminal pro-B-type natriuretic peptide; PVR, pulmonary vascular resistance; PWTd, posterior wall thickness at end-diastole; RVEDd, right ventricular end-diastolic dimension; sST2, soluble suppression of tumorigenicity 2; VKA, Vitamin K antagonists; VO_2_max, maximal oxygen uptake; WBC, white blood cells.

**Table 2:** Univariable and multivariable analysis of predictors related to one-year mortality.

	Univariable Data		Multivariable Data Model 1		Multivariable Data Model 2	
Parameter	HR (95% CI)	*p*	HR (95% CI)	*p*	HR (95% CI)	*p*
CRP ^(+)^	1.018 [1.001–1.035]	0.0353				
Fibrinogen ^(+)^	1.004 [1.003–1.005]	<0.001	1.002 [1.001–1.004]	<0.001	1.002 [1.000–1.003]	0.0099
ALP ^(+)^	1.005 [1.000–1.010]	0.0472				
GGTP ^(+)^	1.006 [1.003–1.009]	<0.001				
Uric acid ^(+)^	1.003 [1.002–1.004]	<0.001	1.001 [1.000–1.002]	0.0426	1.001 [1.000–1.002]	0.0489
Urea ^(+)^	1.023 [0.996–1.051]	0.0980				
NT-proBNP ^(a)^	1.006 [1.003–1.009]	<0.001	1.004 [1.004–1.007]	0.0081	1.005 [1.002–1.008]	0.0020
Sodium ^(−)^	1.164 [1.103–1.229]	<0.001	1.065 [1.004–1.130]	0.0360		
sST2/LVMI-MELD-XI ^(+)^	2.718 [2.369–3.118]	<0.001	2.501 [2.168–2.886]	<0.001		
sST2/LVMI-modMELD ^(+)^	2.718 [2.389–3.092]	<0.001			2.552 [2.224–2.928]	<0.0001

Abbreviations: see Table 1, CI, confidence interval; HR, hazard ratio. ^(+)^ per one unit increase. ^(−)^ per one unit decrease. ^(a)^ per 100-unit increase.

**Table 4:** A comparison of the areas under the ROC curves for the new scales and their components.

	sST2/LVMI-modMELD, AUC [±95 CI] ^1^	*p*
modMELD, AUC [±95 CI]	0.0214 [0.00251–0.0402]	0.0263
sST2/LVMI, AUC [±95 CI]	0.0862 [0.0540–0.1183]	0.0001
	sST2/LVMI-MELD-XI, AUC [±95 CI] ^1^	*p*
MELD-XI, AUC [±95 CI]	0.0486 [0.0213–0.0760]	0.0005
sST2/LVMI AUC [±95 CI]	0.0456 [0.0157–0.0755]	0.0028

^1^ AUC difference between the new scale and its components. Abbreviations: see Table 1; AUC, area under the curve; CI, confidence interval.

## Data Availability

The data presented in this study are available on request from the corresponding author. The data are not publicly available due to privacy restrictions related to the rules in our institution.
